# Income disparities in loss in life expectancy after colon and rectal cancers: a Swedish register-based study

**DOI:** 10.1136/jech-2024-221916

**Published:** 2024-03-21

**Authors:** Elisavet Syriopoulou, Erik Osterman, Alexander Miething, Caroline Nordenvall, Therese Marie-Louise Andersson

**Affiliations:** 1 Department of Medical Epidemiology and Biostatistics, Karolinska Institutet, Stockholm, Sweden; 2 Department of Surgery, Gävle Hospital, Gävle, Sweden; 3 Department of Molecular Medicine and Surgery, Karolinska Institutet, Stockholm, Sweden; 4 Department of Public Health Sciences, Stockholm University, Stockholm, Sweden; 5 Department of Pelvic Cancer, Colorectal Surgery Unit, Karolinska University Hospital, Stockholm, Sweden

**Keywords:** health inequalities, epidemiology, public health, biostatistics

## Abstract

**Background:**

Differences in the prognosis after colorectal cancer (CRC) by socioeconomic position (SEP) have been reported previously; however, most studies focused on survival differences at a particular time since diagnosis. We quantified the lifetime impact of CRC and its variation by SEP, using individualised income to conceptualise SEP.

**Methods:**

Data included all adults with a first-time diagnosis of colon or rectal cancers in Sweden between 2008 and 2021. The analysis was done separately for colon and rectal cancers using flexible parametric models. For each cancer and income group, we estimated the life expectancy in the absence of cancer, the life expectancy in the presence of cancer and the loss in life expectancy (LLE).

**Results:**

We found large income disparities in life expectancy after a cancer diagnosis, with larger differences among the youngest patients. Higher income resulted in more years lost following a cancer diagnosis. For example, 40-year-old females with colon cancer lost 17.64 years if in the highest-income group and 13.68 years if in the lowest-income group. Rectal cancer resulted in higher LLE compared with colon cancer. Males lost a larger proportion of their lives. All patients, including the oldest, lost more than 30% of their remaining life expectancy. Based on the number of colon and rectal cancer diagnoses in 2021, colon cancer results in almost double the number of years lost compared with rectal cancer (24 669 and 12 105 years, respectively).

**Conclusion:**

While our results should be interpreted in line with what individualised income represents, they highlight the need to address inequalities.

**WHAT IS ALREADY KNOWN ON THIS TOPIC**

- Differences in the prognosis after colorectal cancer (CRC) by socioeconomic position have been reported previously; however, most studies focused on survival differences at a particular time since diagnosis.
- Our study fills a literature gap by quantifying the lifetime impact of CRC and its variation by income using register-based data in Sweden.

**WHAT THIS STUDY ADDS**

- We found large income disparities in life expectancy after a CRC diagnosis, with larger disparities among the youngest patients.
- Patients with early-onset CRC (<50 years old) lost more than one-third of their life expectancy.
- Colon cancer resulted in almost double the number of years lost in a typical year compared with rectal cancer (24 669 and 12 105 lost years, respectively).

**HOW THIS STUDY MIGHT AFFECT RESEARCH, PRACTICE OR POLICY**

- Further research is required to improve our understanding of how the disparities arise as this would allow to target modifiable risk factors with relevant health policies.
- Given the increase in CRC incidence among the youngest that has been reported by several studies over the last years, it is important to study further the impact of CRC on younger patients.

## Background

Colorectal cancer (CRC) is the third most common cancer worldwide and the second most common cause of cancer-related deaths.^1^ The overall incidence of CRC decreased in the last years or remained almost constant, but for patients under 50 years old, the incidence continued to increase over time both in Sweden^2^ and other countries.^3–5^ Screening programmes are implemented in some countries; however, these are generally offered to those above 50 years old and could not justify the increase in incidence.

CRC survival has improved substantially in many countries.^6–8^ Nevertheless, several studies have reported disparities in survival by socioeconomic position (SEP).^9–11^ The differences persist independently of the indicator used to conceptualise SEP and also in countries with universal access to healthcare. Only a few studies have looked at the impact of CRC diagnosis on the whole of the remaining lifespan using measures such as the loss in life expectancy (LLE) after a cancer diagnosis.^12 13^ A Swedish study showed substantial losses in life expectancy after a colon cancer diagnosis but did not take SEP into account.^14^ An English study investigated differences in life expectancy by SEP and found a socioeconomic gradient for both colon and rectal cancers.^15^

Exploring disparities in cancer prognosis and identifying population groups with worse outcomes are of high value. In England, eliminating differences in relative survival across all deprivation groups was estimated to result in almost 8231 and 7295 life-years gain based on the number of colon and rectal cancer diagnoses in 2013, respectively.^15^ The magnitude of such gains highlights the importance of further research on cancer inequalities that will improve our understanding and will provide an evidence basis for relevant policies aiming to eliminate socioeconomic differences in cancer survival.

In this study, we quantify the lifetime impact of a colon and rectal cancer diagnosis in Sweden and explore how this differs by SEP using individualised disposable income to conceptualise SEP.

## Methods

### Data resources

Data included all adults diagnosed with a first-time diagnosis of colon or rectal cancers in Sweden between 2008 and 2021 and follow-up time to the end of 2021. Patient data originated from the Colorectal Cancer Database (CRCBaSe), a register linkage of the Swedish Colorectal Cancer Registry (SCRCR) and national registries at the National Board of Welfare, and Statistics Sweden.^16^ There was also information on disposable income per consumption unit for family (variable DispInkKE) that was gathered from the Longitudinal Integrated Database for Health Insurance and Labour Market Studies (LISA). Disposable income per consumption unit for a family is obtained by the sum of the disposable income of all members of the family divided with the consumption weight that applies to the household and is described in more detail elsewhere.^17^ We chose to conceptualise SEP using a household-based income indicator, as this is more appropriate for representing the availability of material resources and corresponding health awareness among individuals with lower individual disposable income but access to more household resources. For instance, when using individual disposable income to allocate patients in SEP groups, housewives with low individual income but with access to a higher household income may be misclassified to lower SEP groups. In our study, the disposable income per consumption unit for a family of each individual was obtained as the average of the 3 years prior to their diagnosis.

### Statistical analysis

Data were analysed separately for colon and rectal cancers. We analysed the data using flexible parametric survival models (FPMs) that have advantages in terms of incorporating complex effects in comparison with traditional models such as Cox models.^18^ In particular, FPMs easily allow incorporation of interactions and non-proportional effects that are common with cancer data. FPMs have also been extended to estimate life expectancy measures which was the main aim of this study^12^ and have been shown to be insensitive to the choice of the degrees of freedom (df).^19 20^ For each cancer, we fitted a FPM that included sex, age (included as a continuous variable in the model using natural splines to allow for a non-linear effect, 3 df) and individualised income (4 groups). The baseline excess hazard was modelled using 5 df. We allowed time-dependent effects for sex, age (continuously using natural splines with 2 df) and income (using 3 df). In this way, their effect is allowed to vary with time since diagnosis. Interactions were also included between sex and income, and income and age at diagnosis to allow for a different effect of income in males and females as well as across age at diagnosis.

We obtained estimates of average relative survival at each year after diagnosis for each income group.^21^ For each income group, we also estimated the average life expectancy in the absence of cancer (general population) and presence of cancer (cancer population), as well as the difference between these two, that is, LLE. LLE corresponds to the average number of life-years lost as a result of a cancer diagnosis, and it was estimated from the time of diagnosis of patients. To enable estimation of life expectancy, extrapolation of the survival curves beyond follow-up time was performed using the method described by Andersson *et al*.^12^

The above estimates represent the actual estimates observed in each income group. Each income group may have a different age distribution, for example, in some groups, there may be more elderly patients. To account for differences in the age distribution, we also obtained age-standardised and sex-standardised estimates over the whole population (using a common age distribution across estimates by income). These provide a fairer comparison between income groups as they are not influenced by differences in the age distribution.

In addition, the proportion of life lost (PLL) and the total years lost (TYL) based on the diagnoses of 2021 were estimated. PLL accounts for the fact that younger patients have more years to lose and is more comparable across ages. It is calculated as the LLE divided by the life expectancy in the general population. TYL was calculated as the LLE multiplied by the number of diagnoses in each income group in 2021. TYL provides an estimate of the impact of cancer on a population level and can be of great interest to quantify the impact of cancer from a public health point of view.

Finally, age-specific estimates of all of the above measures were obtained for males and females, to explore how the lifetime impact of cancer varies by sex, age at diagnosis and income group.

### Lifetables for the general population

For the estimation of relative survival and life expectancy measures, we used population lifetables that included information on expected mortality rates by sex, calendar year, age and income group. These were used as a proxy for the mortality rates of each patient with cancer if they did not have the cancer and were constructed as part of a previous study. Briefly, the lifetables were constructed by adjusting already available lifetables by sex, age and year, obtained from the Human Mortality Database,^22^ to include information on individualised income. The reason for having to stratify the lifetables by income is that both other-cause mortality and cancer mortality vary by income group, which was the exposure of interest in our analysis. Thus, we had to further stratify the population lifetable by income to ensure that we compare patients with similar individuals in the general population. We applied the approach by Bower *et al* and used data on comparators from the general population matched to our cancer population on birth year, sex, year of diagnosis and county.^23^ Separate lifetables were constructed for colon and rectal cancers, using the controls for each cancer. The cut-off points for the income groups were based on the cut-offs of the quartiles for the income distribution of the controls that were available in our dataset and were assumed to be a proxy for the general population. We created quartiles separately for individuals above 65 years old and below 65 years old to deal with potential misclassification of the oldest retired individuals to lower SEP due to lower income compared with the working group. The income categories of our analysis are not of equal size, and we allow SEP groups in our cancer cohort to have different sizes which is in line with what is reported by several studies (incidence will be higher among certain SEP groups^24^).

## Results

The analysis included 58 162 patients with colon cancer and 27 691 patients with rectal cancer. The number of patients diagnosed with colon cancer was similar in males and females, while rectal cancer was more common in males. There were more males diagnosed in the highest income groups, while the opposite was observed for females. More details on descriptive statistics are available in online supplemental table 1.

10.1136/jech-2024-221916.supp1 Supplementary data

### Average and standardised estimates

**Figure 1:**
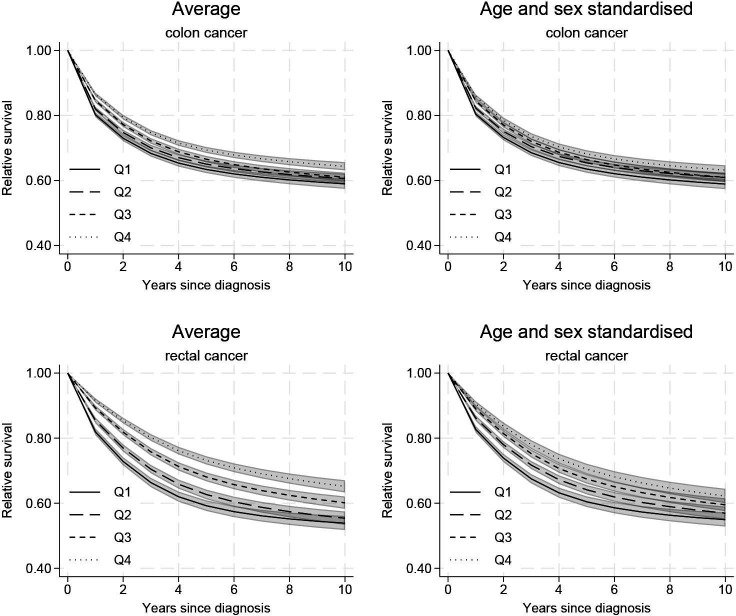
Average relative survival as well as age-standardised and sex-standardised relative survival by income group (from the lowest (Q1) to the highest (Q4) income), with 95% CIs, both for colon and rectal cancers.

There were large differences in average relative survival between income groups. For colon cancer, the absolute difference between the highest-income and lowest-income groups remained above 5% (figure 1, left panel). The equivalent differences were larger for rectal cancer and remained above 9% (figure 1, left panel). Disparities increased with time for rectal cancer but decreased for colon cancer. A part of the observed disparities were driven by differences in the age distribution within income groups. However, disparities remained high even after obtaining age-standardised relative survival estimates to account for age differences (figure 1, right panels).

There were also large differences in life expectancy across income groups. Differences were already present in the general population. In table 1, for instance, individuals in the lowest-income group had a life expectancy of 13.64 years on average, while individuals in the highest-income group had an extra 4 years. Differences were also present among patients with cancer. Colon cancer reduced the life expectancy of individuals from the lowest-income and highest-income groups to 8.81 (95% CI: 8.61 to 9.01) and 12.10 (95% CI: 11.88 to 12.32) years on average, respectively, yielding losses of 4.83 (95% CI: 4.63 to 5.03) and 5.73 (95% CI: 5.51 to 5.94) years for the lowest-income and highest-income groups. Highest-income group lost 1 year more on average. This translates into the lowest-income group losing a higher proportion of their remaining life expectancy following their colon cancer diagnosis, equal to 35% (95% CI: 34 to 37) for lowest-income group and 32% (95% CI: 31 to 33) for the highest. On the population level and based on the number of diagnoses in 2021, colon cancer resulted in 2714 years lost in the lowest and 9320 years lost in the highest-income groups. The larger number of losses in the highest-income group is largely driven by the more patients in that group. For all four income groups, colon cancer resulted in 24 669 years lost.

**Table 1:** Average life expectancy measures as well as age-standardised and sex-standardised estimates by income group (from the lowest (Q1) to the highest (Q4) income) for colon cancer, with 95% CIs in the parentheses. The total years lost are based on the number of colon cancer diagnoses in 2021. Life expectancy measures are estimated from time of diagnosis

Measure	Q1	Q2	Q3	Q4
Average estimates				
Life expectancy in the general population	13.64	13.91	15.36	17.83
Life expectancy in the cancer population	8.81 (8.61 to 9.01)	9.24 (9.06 to 9.42)	10.14 (9.94 to 10.34)	12.10 (11.88 to 12.32)
Loss in life expectancy	4.83 (4.63 to 5.03)	4.68 (4.49 to 4.86)	5.23 (5.03 to 5.42)	5.73 (5.51 to 5.94)
Proportion of life lost	35% (34 to 37)	34% (32 to 35)	34% (33 to 35)	32% (31 to 33)
Total years lost	2714	4952	7683	9320
Age-standardised and sex-standardised estimates				
Life expectancy in the general population	13.80	14.64	15.76	16.35
Life expectancy in the cancer population	8.86 (8.67 to 9.06)	9.69 (9.49 to 9.88)	10.36 (10.16 to 10.58)	11.05 (10.83 to 11.28)
Loss in life expectancy	4.94 (4.74 to 5.13)	4.95 (4.76 to 5.14)	5.40 (5.19 to 5.61)	5.30 (5.08 to 5.53)
Proportion of life lost	36% (34 to 37%)	34% (33 to 35%)	34% (33 to 36%)	32% (31 to 34%)
Total years lost	2775	5245	7935	8628

Rectal cancer had a larger impact on life expectancy, and there were larger differences between income groups (table 2). Following the rectal cancer diagnosis, the lowest-income and highest-income groups lost on average 5.47 (95% CI: 5.16 to 5.76) and 6.02 (95% CI: 5.63 to 6.40) years, respectively. This corresponds to 37% (95% CI: 35 to 39) and 31% (95% CI: 29 to 33) of their remaining life expectancy. On a population level and based on the number of diagnoses in 2021, there was a total of 12 105 years lost following a rectal cancer diagnosis across all groups. This is lower to the equivalent number for colon cancer despite the higher LLE following rectal cancer and is driven by the fewer annual diagnoses of rectal cancer. When estimating age-standardised and sex-standardised estimates to account for age differences, disparities between income groups remained but were reduced (tables 1 and 2).

**Table 2:** Average life expectancy measures as well as age-standardised and sex-standardised estimates by income quartile (from the lowest (Q1) to the highest (Q4) income) for rectal cancer, with 95% CIs in the parentheses. The total years lost are based on the number of rectal cancer diagnoses in 2021. Life expectancy measures are estimated from the time of diagnosis

Measure	Q1	Q2	Q3	Q4
Average estimates				
Life expectancy in the general population	14.69	15.58	17.62	19.63
Life expectancy in the cancer population	9.22 (8.92 to 9.53)	10.15 (9.85 to 10.45)	11.70 (11.37 to 12.04)	13.61 (13.23 to 14.01)
Loss in life expectancy	5.47 (5.16 to 5.76)	5.43 (5.12 to 5.73)	5.92 (5.58 to 6.25)	6.02 (5.63 to 6.40)
Proportion of life lost	37% (35 to 39)	35% (33 to 37)	34% (32 to 35)	31% (29 to 33%)
Total years lost	1492	2563	3511	4539
Age-standardised and sex-standardised estimates				
Life expectancy in the general population	15.07	16.44	17.64	18.14
Life expectancy in the cancer population	9.45 (9.16 to 9.76)	10.73 (10.41 to 11.05)	11.65 (11.31 to 12.00)	12.48 (12.09 to 12.88)
Loss in life expectancy	5.62 (5.31 to 5.92)	5.71 (5.38 to 6.02)	6.00 (5.64 to 6.34)	5.65 (5.25 to 6.04)
Proportion of life lost	37% (35 to 39)	35% (33 to 37)	34% (32 to 36)	31% (29 to 33)
Total years lost	1534	2695	3556	4263

### Age-specific estimates by sex

**Figure 2:**
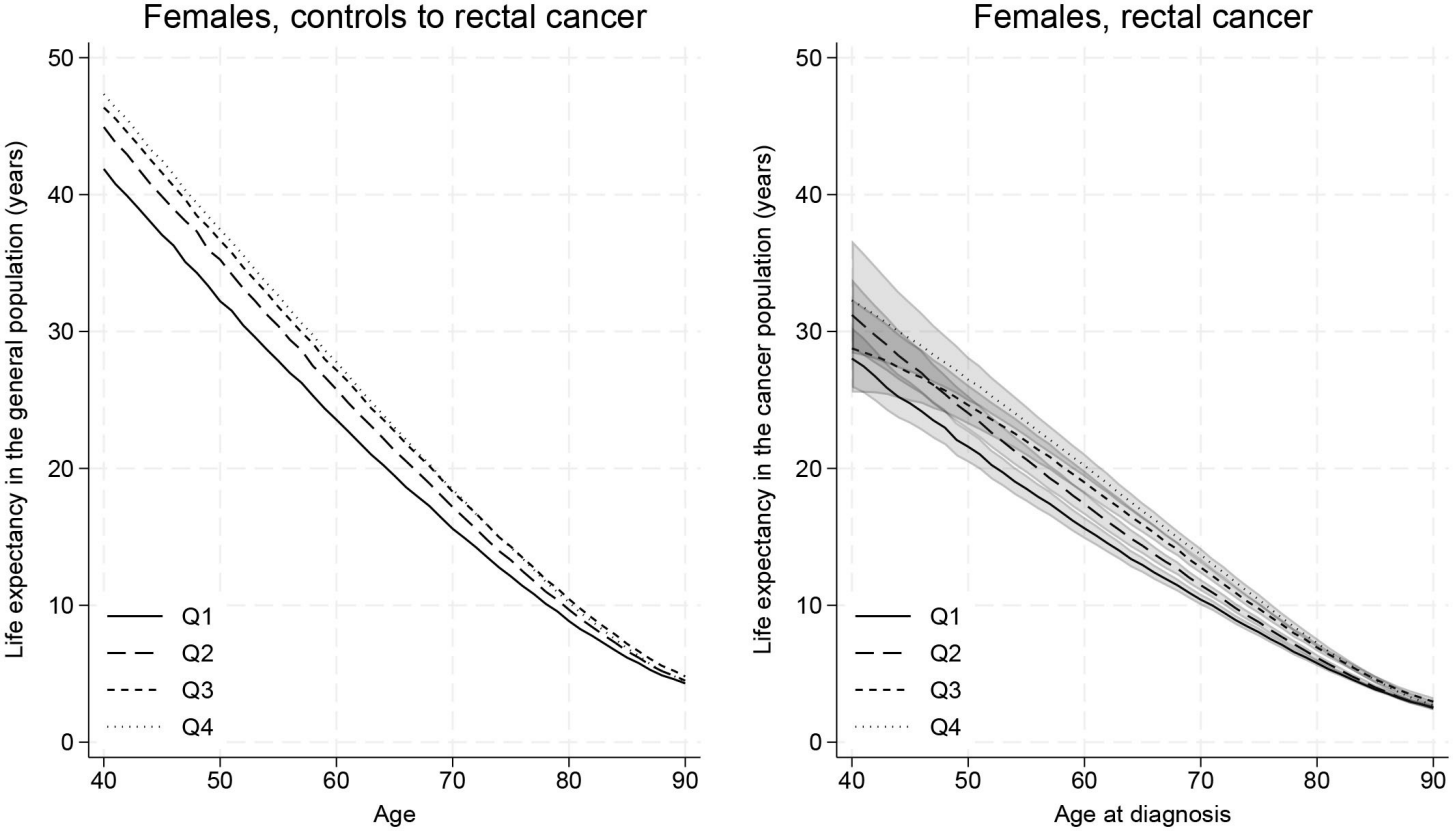
Colon cancer: age-specific life expectancy with and without colon cancer for females by income group (from the lowest (Q1) to the highest (Q4) income), with 95% CIs. Life expectancy measures are estimated from time of diagnosis.

**Figure 3:**
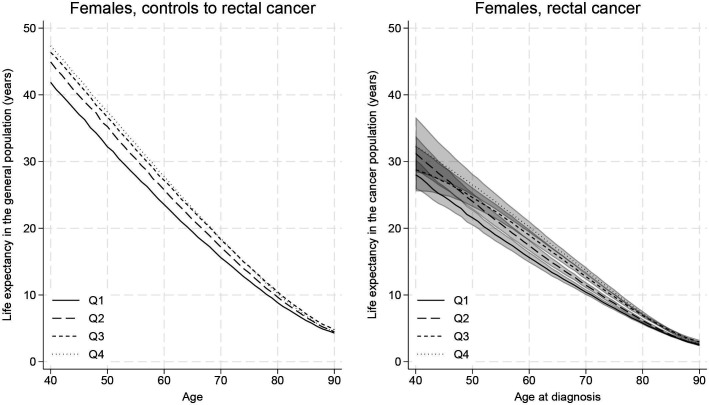
Rectal cancer: age-specific life expectancy with and without rectal cancer for females by income group (from the lowest (Q1) to the highest (Q4) income), with 95% CIs. Life expectancy measures are estimated from the time of diagnosis.

The measures described above vary substantially by age at diagnosis. Younger individuals had a longer life expectancy (figures 2 and 3, online supplemental figures 1 and 2), and they lost more years following their cancer diagnosis (online supplemental figures 3 and 4). For instance, males with colon cancer from the highest-income group lost 19.41 (95% CI: 16.24 to 22.23) years if diagnosed at 40 years old and 2.64 (95% CI: 2.48 to 2.80) years if diagnosed at 80 years old (online supplemental table 2). Although the higher LLE for younger patients is partly due to them having more years to lose to begin with, they still lost a higher proportion of their remaining life expectancy compared with the oldest patients. In the previous example with male patients with colon cancer from the highest-income group, the 40-year-old patient lost 43% (95% CI: 36 to 49) and the 80-year-old patient lost 29% (95% CI: 28 to 31) of their remaining life expectancy. Older individuals were still, however, losing a substantial proportion of their remaining life expectancy as a result of their diagnosis for both colon and rectal cancer; for example, 44% (95% CI: 39 to 48) of the remaining life expectancy lost for 90-year-old males from the lowest-income group with rectal cancer (online supplemental table 4).

Females in the control population for rectal cancer in the two highest-income groups had similar life expectancy. Nevertheless, females in the second highest-income group lost more years (figure 3 and online supplemental figure 4). For instance, for females diagnosed at 40 years old, the highest-income group had a loss of 15.07 (95% CI: 10.68 to 18.94) years while the second highest group lost 17.60 (95% CI:13.98 to 20.82) years (online supplemental table 5). Males lost a higher proportion of their life expectancy compared with females for both cancers.

When comparing life expectancy across different income groups, those with the highest income lost more years but a smaller proportion of their remaining life expectancy, given a higher background life expectancy. However, the youngest individuals from the highest-income group lost a higher proportion of life (for colon cancer) or similar proportion (for rectal cancer) compared with similar individuals from the lowest-income groups. As an example, 40-year-old females with colon cancer lost 17.64 (95% CI:14.57 to 20.42) years (ie, 37% (95% CI: 31 to 43) of their life) if in the highest-income group and 13.68 (95% CI: 12.06 to 15.20) years (ie, 32% (95% CI: 29 to 36) of their life) if in the lowest income group (online supplemental table 3). Income disparities persisted across all ages, but overall, disparities were smaller for older individuals.

## Discussion

In this nationwide study, we found that LLE after a CRC diagnosis substantially differed by income group. Patients in the highest-income group lost more years than those in the lowest-income group. For both males and females and for all income groups, patients with early-onset CRC (<50 years at diagnosis) lost more than one-third of their life expectancy following their cancer diagnosis.

We found large differences in relative survival of more than 5 percentage points for colon and more than 9 percentage points for rectal cancer, with the lowest-income groups having worse prognosis. Differences in age distribution explain part of the gap but not entirely, as age-standarised and sex-standardised estimates varied substantially between income groups. We also found large disparities in life expectancy after a cancer diagnosis between income groups, with larger differences among the youngest. Higher income resulted in more years lost following a diagnosis, partly driven by a longer life expectancy also in the general population, but we found a decreasing PLL with increasing income. Males lost a larger proportion of their lives compared with females, and rectal cancer resulted in a higher LLE compared with colon cancer. All patients lost a substantial part of their remaining life expectancy (more than 30%). Overall, younger individuals lose more years as they have more years to lose. Colon cancer resulted in almost double the number of years lost compared with rectal cancer in a typical year.

Several studies have reported an increase in the incidence of CRC in individuals younger than 50 years old, over the last years.^2 3 25^ An especially relevant finding of our study is that for the youngest individual (eg, 40 years at diagnosis), patients from all income groups lose a substantial proportion of their remaining life expectancy, with patients from the highest-income groups losing as much as 43% of their life. A recent study of early-onset CRC (EOCRC) from high-income countries in Asia and Middle East showed that prognosis following EOCRC has improved more for males than females measured by mortality-incidence ratio.^25^ These results suggest that further research should focus on understanding the impact of CRC on younger patients. Generally, in CRC, females have a lower mortality than males, and in the present study, the female patients had a lower PLE than the males. Differences between males and females have been reported by other studies as well, and the observed differences are most likely the result of multiple factors.^26^

The reasons for the income gradient observed among income groups are likely multifactorial. Part of these disparities could be driven by differences at the stage of diagnosis. However, even though screening programmes are available for CRC in other countries, during the period of our study, no screening programme had been fully implemented in Sweden. Thus, our results cannot be explained by differences in screening attendance. However, potential differences in stage at diagnosis could be driven by differences in health awareness between patients from different income groups.

Another potential factor that can explain part of the differences between income groups is treatment allocation. A recent Swedish study of non-metastasised CRC described that income was associated with what treatment the patients received, despite almost all patients being discussed in multidisciplinary meetings.^27^ Although there are guidelines on how to treat patients with CRC, differences have been reported before. For instance, a study in Sweden that explored clinical decision-making by interviewing oncologists reported that treatment could be tailored for patients living alone to avoid harmful side effects and that patients with the highest SEP could be overtreated.^28^ Differences in timing to start treatment could also explain part of the disparities. Another potential factor is the presence of comorbidities that preclude the allocation of aggressive treatment to older patients or those with underlying conditions.

In our study, we used the individualised income to conceptualise SEP, and our results should be interpreted in line with what individualised income represents.^29^ It is also important to acknowledge that income and income position might be influenced by earlier health problems. Even though access to healthcare is equally available to all Swedish residents and almost for free, we believe that income acts as a good proxy for a person’s access to a healthy lifestyle and health awareness. It captures paths that affect cancer outcomes through someone’s relative position and integration into society as well as the easiness of navigating the healthcare system.^29–31^ Several types of income may be considered to represent SEP. As individual disposable income can be problematic for individuals with low individual income but with access to other household resources, such as older individuals and females, we used a household-based income indicator instead. Another indicator that may be used to conceptualise SEP is education. A potential limitation with education-based indicators is the changes in the school system, and education level could be limited for the older individuals who might have left school immediately after compulsory schooling. Occupation-based indicators for SEP could also be limited for older retired individuals that are no longer part of the working force but who consist of a substantial part of our study population.

A recent study in England and Wales explored the association between cancer survival with area-level deprivation and individual-level deprivation (using indicators of occupation, education and income).^32^ The authors reported excess hazard ratios and 5-year net survival and found persistent area-level deprivation inequalities for CRC even after adjusting for individual socioeconomic variables and highlighted the importance of individual-level indicators of deprivation as these cannot be replaced by area-based measures of deprivation alone. Another study, also in England and Wales, investigated the association between individual-level socioeconomic status and CRC survival and found that both individual-level and area-based indicators of deprivation were associated with survival, highlighting the complex mechanisms in the relationship between socioeconomic factors and cancer outcomes.^33^

A strength of our study is that we used population-based data with everyone diagnosed with CRC in Sweden over a long follow-up period to the end of 2021. We quantified the lifetime impact of CRC instead of looking only at one particular timepoint as in traditional measures such as 5-year relative survival, and we provided a range of measures such as PLL and TYL that are useful from a public health point of view. When interpreting the differences in the years lost between income groups, it is important to also consider differences in the life expectancy with and without cancer. For instance, a higher loss in life years for a certain income group compared with another could be the result of that group having more years to lose (higher life expectancy even in the absence of cancer); it could be the result of a more substantial impact of cancer on life expectancy or both.

We found a large gap in the impact of a colon and rectal cancer diagnosis on life expectancy by income group. Further research is required to disentangle the drivers of the observed disparities as this would allow to improve our understanding of how the differences arise and will help to target modifiable risk factors. For instance, if survival differences between the least and most deprived are driven by differences in stage at diagnosis, health policies could be implemented to encourage earlier detection in groups with worse survival and ultimately reduce health inequalities in society. If differential treatment allocation is partly responsible for the observed disparities, it is crucial to implement stricter clinical guidelines for the cancer management and care. Future work will focus on exploring the underlying determinants of the observed disparities using mediation analysis methods.

## Data Availability

Data may be obtained from a third party and are not publicly available. This study analysed data from the CRCBaSe. Restrictions apply to the availability of these data and so are not publicly available. However, data requests can be send to the relevant quality registry for CRC: https://cancercentrum.se/samverkan/cancerdiagnoser/tjocktarm-andtarm-och-anal/tjock--och-andtarm/kvalitetsregister/forskning/forskningsdatabas/.
