# Supplementary material for: Income disparities in loss in life expectancy after colon and rectal cancers: a Swedish register-based study
Source: J Epidemiol Community Health. 2024 Mar 21;78(6):402–8. doi: 10.1136/jech-2024-221916 (PMC11103304; doi:10.1136/jech-2024-221916)
Supplement: Supplementary data [file jech-2024-221916supp001.pdf]

Supplementary material

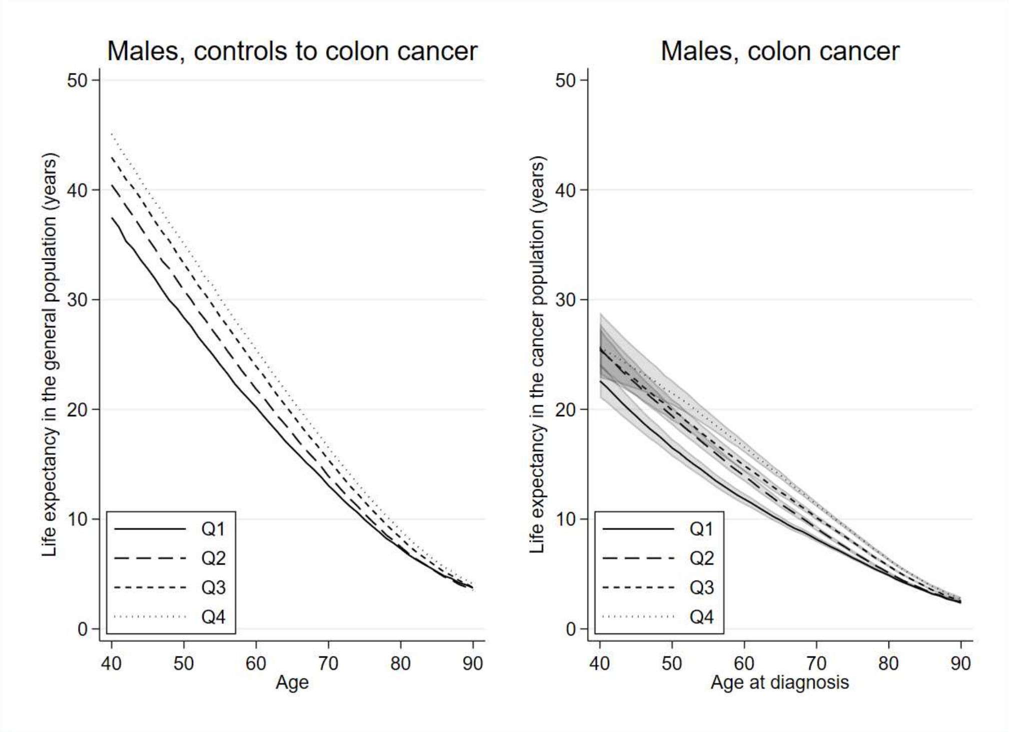

Supplementary Figure 1: Colon cancer: age-specific life expectancy with and without colon cancer for males by income group (from the lowest (Q1) to the highest (Q4) income), with 95% confidence intervals.

Syriopoulou E, *et al. J Epidemiol Community Health* 2024; 78:402–408. doi: 10.1136/jech-2024-221916

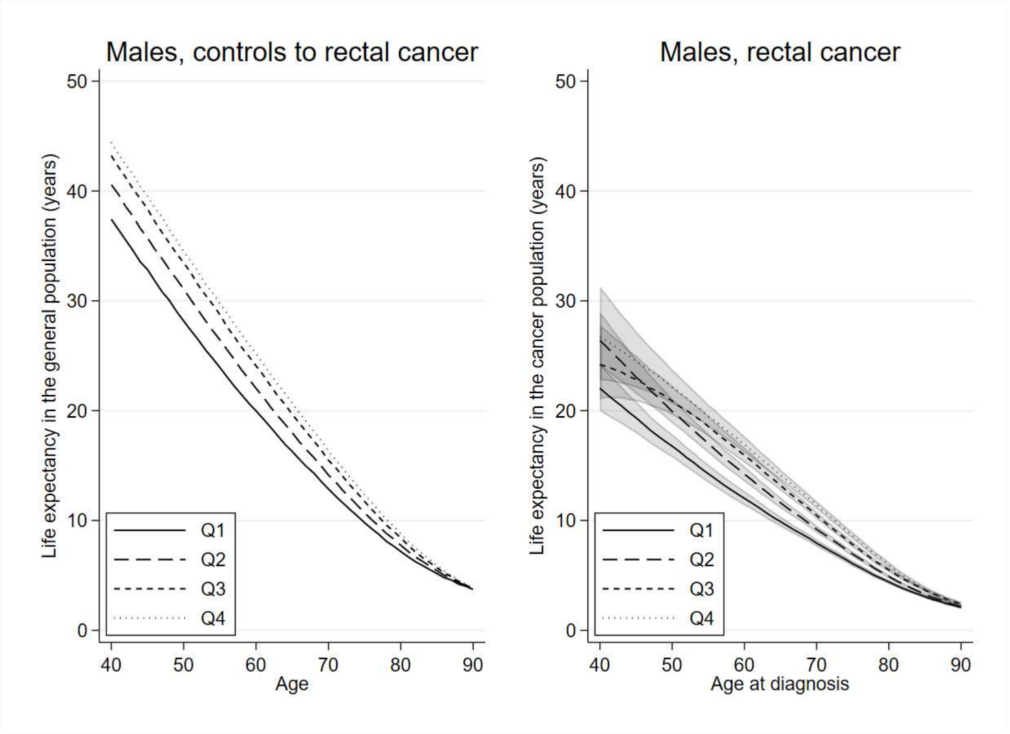

Supplementary Figure 2: Rectal cancer: age-specific life expectancy with and without rectal cancer for males by income group (from the lowest (Q1) to the highest (Q4) income), with 95% confidence intervals.

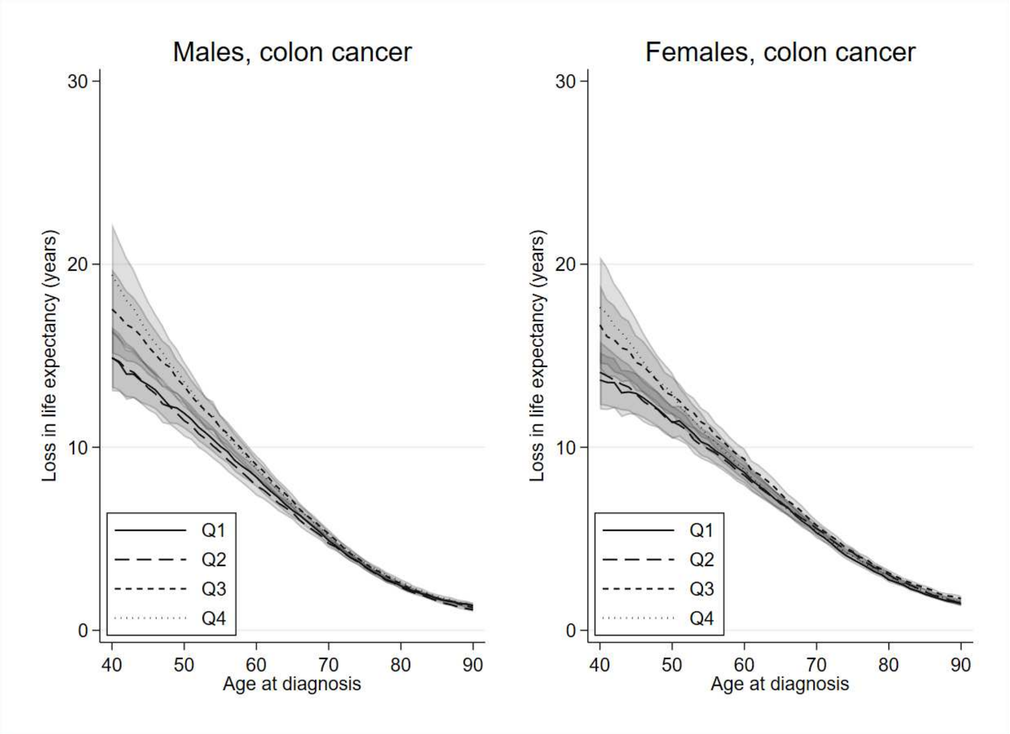

Supplementary Figure 3: Colon cancer: age-specific loss in life expectancy after a colon cancer diagnosis by income group (from the lowest (Q1) to the highest (Q4) income) for males and females, with 95% confidence intervals.

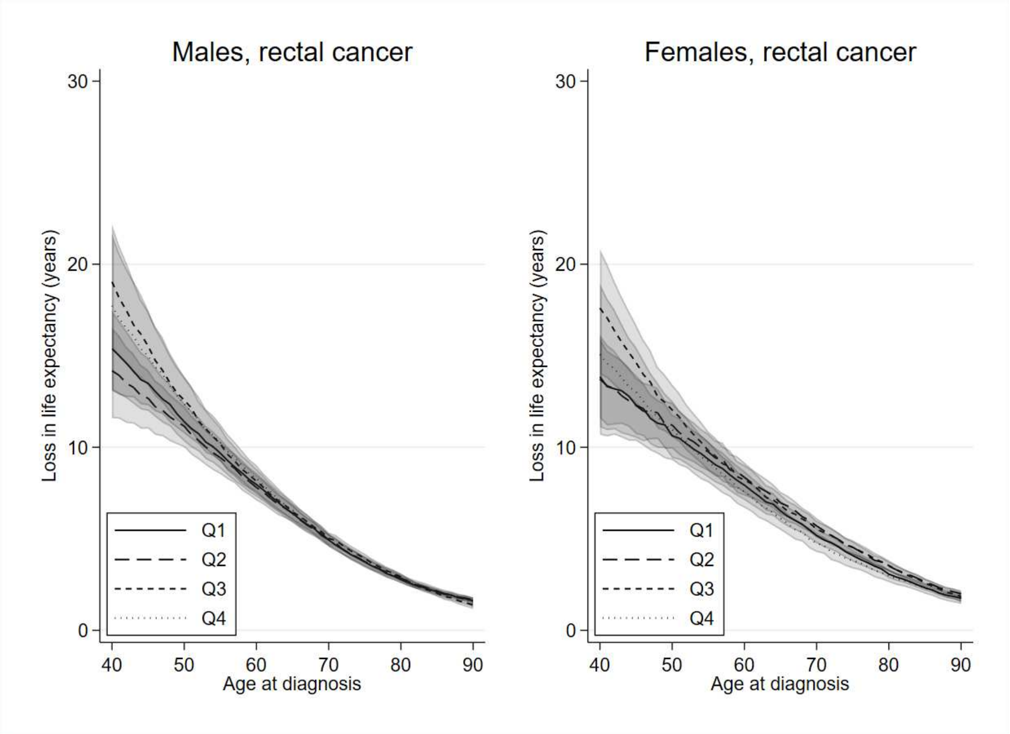

Supplementary Figure 4: Rectal cancer: age-specific loss in life expectancy after a rectal cancer diagnosis by income group (from the lowest (Q1) to the highest (Q4) income) for males and females, with 95% confidence intervals.

Syriopoulou E, *et al. J Epidemiol Community Health* 2024; 78:402–408. doi: 10.1136/jech-2024-221916

Supplementary Table 1: Descriptive statistics for the colon and cancer patients by income group and sex.

Cancer	Sex	Income group	Number of patients	Median age (in years)
Colon	Males	1	4910	74
	Males	2	6440	76
	Males	3	8421	75
	Males	4	9016	70
	Females	1	7350	78
	Females	2	8147	77
	Females	3	7247	74
	Females	4	6631	70
Rectal	Males	1	3083	72
	Males	2	4070	74
	Males	3	4647	71
	Males	4	4882	68
	Females	1	2953	77
	Females	2	2922	74
	Females	3	2654	70
	Females	4	2480	67

Supplementary Table 2: Age-specific estimates of life expectancy (LE in years) and loss in life expectancy (LLE in years) and proportion of life lost (PLL) after a colon cancer diagnosis by income quartile (from the lowest (Q1) to the highest (Q4) income) for males, with 95% confidence intervals in the parentheses.

	Q1				Q2				Q3				Q4			
Age at diagnosis	LE w/t cancer	LE with cancer	LLE	PLL	LE w/t cancer	LE with cancer	LLE	PLL	LE w/t cancer	LE with cancer	LLE	PLL	LE w/t cancer	LE with cancer	LLE	PLL
40	37.49	22.59 (21.06 - 24.23)	14.90 (13.26 - 16.43)	40% (35% - 44%)	40.45	25.57 (23.88 - 27.38)	14.87 (13.06 - 16.57)	37% (32% - 41%)	42.95	25.42 (23.21 - 27.83)	17.54 (15.12 - 19.74)	41% (35% - 46%)	45.09	25.69 (22.86 - 28.86)	19.41 (16.24 - 22.23)	43% (36% - 49%)
50	28.35	16.49 (15.68 - 17.33)	11.86 (11.02 - 12.66)	42% (39% - 45%)	30.84	19.40 (18.56 - 20.27)	11.45 (10.57 - 12.28)	37% (34% - 40%)	33.27	19.92 (18.95 - 20.93)	13.35 (12.33 - 14.32)	40% (37% - 43%)	35.07	21.50 (20.39 - 22.67)	13.56 (12.39 - 14.68)	39% (35% - 42%)
60	20.19	11.83 (11.31 - 12.36)	8.37 (7.83 - 8.88)	41% (39% - 44%)	21.83	13.94 (13.42 - 14.48)	7.89 (7.35 - 8.41)	36% (34% - 39%)	23.90	14.87 (14.34 - 15.41)	9.03 (8.48 - 9.56)	38% (35% - 40%)	25.41	16.56 (16.05 - 17.08)	8.85 (8.33 - 9.36)	35% (33% - 37%)
70	13.05	8.15 (7.89 - 8.43)	4.90 (4.63 - 5.16)	38% (35% - 40%)	13.90	9.14 (8.89 - 9.40)	4.75 (4.49 - 5.00)	34% (32% - 36%)	15.39	10.13 (9.88 - 10.38)	5.27 (5.01 - 5.51)	34% (33% - 36%)	16.49	11.33 (11.09 - 11.58)	5.16 (4.91 - 5.40)	31% (30% - 33%)
80	7.31	4.89 (4.74 - 5.04)	2.42 (2.27 - 2.57)	33% (31% - 35%)	7.46	5.10 (4.97 - 5.23)	2.36 (2.23 - 2.49)	32% (30% - 33%)	8.28	5.73 (5.60 - 5.86)	2.55 (2.42 - 2.68)	31% (29% - 32%)	8.94	6.30 (6.15 - 6.47)	2.64 (2.48 - 2.80)	29% (28% - 31%)
90	3.73	2.37 (2.26 - 2.49)	1.35 (1.24 - 1.46)	36% (33% - 39%)	3.53	2.41 (2.31 - 2.51)	1.12 (1.01 - 1.22)	32% (29% - 35%)	3.75	2.52 (2.40 - 2.64)	1.24 (1.11 - 1.36)	33% (30% - 36%)	4.08	2.73 (2.56 - 2.92)	1.34 (1.16 - 1.51)	33% (29% - 37%)

Supplementary Table 3: Age-specific estimates of life expectancy (LE in years) and loss in life expectancy (LLE in years) and proportion of life lost (PLL) after a colon cancer diagnosis by income quartile (from the lowest (Q1) to the highest (Q4) income) for females, with 95% confidence intervals in the parentheses.

	Q1				Q2				44				Q4			
Age at diagnosis	LE w/t cancer	LE with cancer	LLE	PLL	LE w/t cancer	LE with cancer	LLE	PLL	LE w/t cancer	LE with cancer	LLE	PLL	LE w/t cancer	LE with cancer	LLE	PLL
40	42.20	28.52 (26.99 - 30.14)	13.68 (12.06 - 15.20)	32% (29% - 36%)	44.23	30.13 (28.44 - 31.92)	14.10 (12.31 - 15.79)	32% (28% - 36%)	46.40	29.72 (27.52 - 32.11)	16.68 (14.30 - 18.88)	36% (31% - 41%)	47.16	29.53 (26.75 - 32.59)	17.64 (14.57 - 20.42)	37% (31% - 43%)
50	32.61	21.26 (20.39 - 22.16)	11.36 (10.45 - 12.23)	35% (32% - 37%)	34.58	23.17 (22.30 - 24.07)	11.41 (10.51 - 12.28)	33% (30% - 36%)	36.51	23.68 (22.66 - 24.75)	12.83 (11.76 - 13.86)	35% (32% - 38%)	37.42	24.45 (23.33 - 25.63)	12.97 (11.79 - 14.09)	35% (32% - 38%)
60	24.00	15.36 (14.78 - 15.97)	8.63 (8.02 - 9.22)	36% (33% - 38%)	25.41	16.94 (16.38 - 17.52)	8.47 (7.88 - 9.03)	33% (31% - 36%)	27.08	17.72 (17.14 - 18.32)	9.36 (8.76 - 9.94)	35% (32% - 37%)	27.67	18.89 (18.34 - 19.46)	8.78 (8.21 - 9.33)	32% (30% - 34%)
70	15.94	10.61 (10.32 - 10.92)	5.32 (5.02 - 5.62)	33% (32% - 35%)	16.97	11.41 (11.12 - 11.70)	5.56 (5.27 - 5.85)	33% (31% - 34%)	18.05	12.32 (12.03 - 12.63)	5.72 (5.42 - 6.02)	32% (30% - 33%)	18.46	13.03 (12.75 - 13.32)	5.43 (5.14 - 5.71)	29% (28% - 31%)
80	9.00	6.25 (6.10 - 6.40)	2.76 (2.61 - 2.90)	31% (29% - 32%)	9.61	6.58 (6.43 - 6.74)	3.03 (2.87 - 3.18)	32% (30% - 33%)	10.33	7.20 (7.03 - 7.38)	3.13 (2.95 - 3.30)	30% (29% - 32%)	10.37	7.36 (7.16 - 7.55)	3.01 (2.82 - 3.20)	29% (27% - 31%)
90	4.27	2.82 (2.71 - 2.93)	1.45 (1.34 - 1.57)	34% (31% - 37%)	4.53	3.05 (2.92 - 3.18)	1.48 (1.35 - 1.61)	33% (30% - 36%)	4.95	3.22 (3.06 - 3.39)	1.73 (1.56 - 1.90)	35% (31% - 38%)	4.55	3.03 (2.84 - 3.23)	1.52 (1.32 - 1.71)	33% (29% - 38%)

Supplementary Table 4: Age-specific estimates of li expectancy (LE in years) and loss in life expectancy (LLE in years) and proportion of life lost (PLL) after a rectal cancer diagnosis by income quartile (from the lowest (Q1) to the highest (Q4) income) for males, with 95% confidence intervals in the parentheses.

	Q1				Q2				Q3				Q4			
Age at diagnosis	LE w/t cancer	LE with cancer	LLE	PLL	LE w/t cancer	LE with cancer	LLE	PLL	LE w/t cancer	LE with cancer	LLE	PLL	LE w/t cancer	LE with cancer	LLE	PLL
40	37.43	22.04 (19.96 - 24.35)	15.38 (13.08 - 17.47)	41% (35% - 47%)	40.58	26.40 (24.04 - 28.98)	14.18 (11.59 - 16.54)	35% (29% - 41%)	43.22	24.18 (21.05 - 27.78)	19.04 (15.45 - 22.17)	44% (36% - 51%)	44.43	26.70 (22.77 - 31.31)	17.73 (13.12 - 21.66)	40% (30% - 49%)
50	28.17	16.77 (15.75 - 17.85)	11.40 (10.31 - 12.42)	40% (37% - 44%)	31.07	19.92 (18.83 - 21.07)	11.15 (10.00 - 12.24)	36% (32% - 39%)	33.47	20.90 (19.61 - 22.26)	12.57 (11.20 - 13.86)	38% (33% - 41%)	34.51	22.14 (20.64 - 23.76)	12.36 (10.75 - 13.87)	36% (31% - 40%)
60	19.98	12.01 (11.38 - 12.68)	7.97 (7.30 - 8.60)	40% (37% - 43%)	22.03	14.22 (13.54 - 14.94)	7.80 (7.09 - 8.48)	35% (32% - 39%)	24.08	15.93 (15.25 - 16.64)	8.14 (7.44 - 8.82)	34% (31% - 37%)	25.22	16.91 (16.18 - 17.67)	8.30 (7.54 - 9.03)	33% (30% - 36%)
70	12.88	7.94 (7.62 - 8.27)	4.94 (4.60 - 5.26)	38% (36% - 41%)	14.14	9.20 (8.88 - 9.53)	4.94 (4.61 - 5.26)	35% (33% - 37%)	15.49	10.47 (10.14 - 10.80)	5.02 (4.69 - 5.35)	32% (30% - 35%)	16.31	11.39 (11.04 - 11.76)	4.92 (4.56 - 5.27)	30% (28% - 32%)
80	7.18	4.40 (4.21 - 4.61)	2.77 (2.57 - 2.96)	39% (36% - 41%)	7.75	4.90 (4.72 - 5.08)	2.85 (2.67 - 3.03)	37% (34% - 39%)	8.43	5.51 (5.31 - 5.71)	2.92 (2.71 - 3.12)	35% (32% - 37%)	8.80	5.97 (5.72 - 6.23)	2.84 (2.58 - 3.09)	32% (29% - 35%)
90	3.72	2.09 (1.94 - 2.26)	1.62 (1.45 - 1.78)	44% (39% - 48%)	3.79	2.13 (1.98 - 2.30)	1.65 (1.49 - 1.81)	44% (39% - 48%)	3.76	2.38 (2.17 - 2.61)	1.38 (1.16 - 1.59)	37% (31% - 42%)	3.78	2.24 (2.00 - 2.51)	1.54 (1.27 - 1.79)	41% (34% - 47%)

Supplementary Table 5: Age-specific estimates of life expectancy (LE in years) and loss in life expectancy (LLE in years) and proportion of life lost (PLL) after a rectal cancer diagnosis by income quartile (from the lowest (Q1) to the highest (Q4) income) for females, with 95% confidence intervals in the parentheses.

	Q1				Q2				Q3				Q4			
Age at diagnosis	LE w/t cancer	LE with cancer	LLE	PLL	LE w/t cancer	LE with cancer	LLE	PLL	LE w/t cancer	LE with cancer	LLE	PLL	LE w/t cancer	LE with cancer	LLE	PLL
40	41.88	28.02 (25.91 - 30.30)	13.85 (11.57 - 15.96)	33% (28% - 38%)	44.94	31.21 (28.79 - 33.84)	13.72 (11.09 - 16.15)	31% (25% - 36%)	46.36	28.76 (25.54 - 32.39)	17.60 (13.98 - 20.82)	38% (30% - 45%)	47.34	32.26 (28.40 - 36.65)	15.07 (10.68 - 18.94)	32% (23% - 40%)
50	32.21	21.58 (20.43 - 22.80)	10.63 (9.41 - 11.78)	33% (29% - 37%)	35.26	24.04 (22.82 - 25.33)	11.22 (9.93 - 12.44)	32% (28% - 35%)	36.65	24.62 (23.22 - 26.11)	12.03 (10.55 - 13.44)	33% (29% - 37%)	37.45	26.48 (24.90 - 28.15)	10.98 (9.31 - 12.55)	29% (25% - 34%)
60	23.56	15.62 (14.85 - 16.44)	7.93 (7.12 - 8.71)	34% (30% - 37%)	25.77	17.41 (16.60 - 18.26)	8.36 (7.51 - 9.18)	32% (29% - 36%)	27.19	18.98 (18.16 - 19.83)	8.21 (7.36 - 9.03)	30% (27% - 33%)	27.78	20.21 (19.38 - 21.08)	7.57 (6.70 - 8.40)	27% (24% - 30%)
70	15.59	10.41 (10.00 - 10.84)	5.18 (4.75 - 5.59)	33% (30% - 36%)	17.17	11.47 (11.03 - 11.92)	5.70 (5.25 - 6.14)	33% (31% - 36%)	18.30	12.77 (12.31 - 13.25)	5.53 (5.05 - 5.99)	30% (28% - 33%)	18.46	13.70 (13.24 - 14.19)	4.76 (4.27 - 5.23)	26% (23% - 28%)
80	8.85	5.78 (5.55 - 6.01)	3.07 (2.84 - 3.30)	35% (32% - 37%)	9.68	6.17 (5.92 - 6.43)	3.51 (3.25 - 3.76)	36% (34% - 39%)	10.45	6.92 (6.62 - 7.22)	3.54 (3.23 - 3.83)	34% (31% - 37%)	10.23	7.25 (6.92 - 7.59)	2.98 (2.64 - 3.31)	29% (26% - 32%)
90	4.30	2.55 (2.38 - 2.72)	1.75 (1.58 - 1.91)	41% (37% - 45%)	4.50	2.50 (2.31 - 2.71)	1.99 (1.79 - 2.19)	44% (40% - 49%)	4.81	2.96 (2.68 - 3.28)	1.84 (1.53 - 2.13)	38% (32% - 44%)	4.44	2.68 (2.38 - 3.01)	1.77 (1.43 - 2.06)	40% (32% - 46%)
